# Isolation of Neuroprotective Anthocyanins from Black Chokeberry (*Aronia melanocarpa*) against Amyloid-β-Induced Cognitive Impairment

**DOI:** 10.3390/foods10010063

**Published:** 2020-12-29

**Authors:** Haichao Wen, Hui Cui, Hehe Tian, Xiaoxu Zhang, Liyan Ma, Charles Ramassamy, Jingming Li

**Affiliations:** 1 Center for Viticulture and Enology, College of Food Science and Nutritional Engineering, China Agricultural University, Beijing 100083, China; wenhc@cau.edu.cn (H.W.); cuihuidaisy@163.com (H.C.); tianhehe@cau.edu.cn (H.T.); 2 College of Food Science and Nutritional Engineering, China Agricultural University, Beijing 100083, China; zxxjoypeace@foxmail.com (X.Z.); lyma1203@cau.edu.cn (L.M.); 3 Supervision, Inspection & Testing Center for Agricultural Products Quality, Ministry of Agriculture, Beijing 100083, China; 4 Institut National de la Recherche Scientifique-Institut Armand Frappier, Laval, QC H7V 1B7, Canada; Ramassamy@iaf.inrs.cn

**Keywords:** black chokeberry, anthocyanin, simulated moving bed, antioxidant activity, neuroprotection, Alzheimer’s disease

## Abstract

Black chokeberry (*Aronia melanocarpa*) fruits are rich in anthocyanins, which are vital secondary metabolites that possess antioxidative properties. The aim of this study was to isolate and purify the anthocyanins from black chokeberry by simulated moving bed (SMB) chromatography, and to investigate the neuroprotective effect of SMB purified anthocyanin against Aβ-induced memory damage in rats. The parameters of the SMB process were studied and optimized. Anthocyanin extracts were identified by HPLC and UPLC-QTOF-MS, and antioxidant abilities were evaluated. The Aβ-induced animal model was established by intracerebral ventricle injection in rat brain. Through the SMB purification, anthocyanins were purified to 85%; cyanidin 3-*O*-galactoside and cyanidin 3-*O*-arabinoside were identified as the main anthocyanins by UPLC-QTOF-MS. The SMB purified anthocyanins exhibited higher DPPH and ABTS free radical scavenging abilities than the crude anthocyanins extract. Furthermore, rats receiving SMB purified anthocyanins treatment (50 mg/kg) showed improved spatial memory in a Morris water maze test, as well as protection of the cells in the hippocampus against Aβ toxicity. These results demonstrate that anthocyanins could serve as antioxidant and neuroprotective agents, with potential in the treatment of Alzheimer’s disease.

## 1. Introduction

Black chokeberry (*Aronia melanocarpa*) is used as an ornamental plant and as a food and colorant. It is rich in the secondary metabolites such as anthocyanins and flavonoids which play vital roles in protecting against oxidative stress and biotic stress [1]. The main anthocyanins in the black chokeberry are cyanidin 3-*O*-galactoside, cyanidin 3-*O*-arabinoside, cyanidin 3-*O*-glucoside, and cyanidin 3-*O*-xyloside. These compounds exhibit many bioactivities such as antioxidant, antiproliferative, antimicrobial, anti-inflammation, and modulate hepatic lipid metabolism activities [1,2,3,4]. Meanwhile, anthocyanins have been shown to prevent and remedy diseases such as cardiovascular disease, liver failure, obesity, and diabetes [5]. Moreover, anthocyanins can cross the blood-brain barrier (BBB) and delay aging-related degenerative diseases [6,7,8]. However, the stability of anthocyanins is influenced by many factors such as structure, the presence of solvents, pH, temperature, oxygen, and enzymes and other concomitant substances; as such it is still impossible to isolate and purify monomeric anthocyanin from complex natural compounds [9].

Neurodegenerative disorders are becoming more and more prevalent, leading to living and economic burdens on the family members of affected individuals. Alzheimer’s disease (AD), one of the most common causes of dementia, is associated with many risk factors including alcohol use, smoking, hypertension, exposure to metals, and oxidative stress [10]. A hallmark of AD is the accumulation of insoluble forms of amyloid-β (Aβ) in the plaques in extracellular spaces and in the walls of blood vessels [10]. Although the pathogenesis of AD is not fully understood, a great deal of research has supported the hypothesis that reactive oxygen species (ROS) impair antioxidant defense systems and induce neuron apoptosis [11]. Many secondary metabolites, like phenolic compounds from Ginkgo biloba, green tea, curcumin, grape, and blueberry, have been reported to protect neuronal cells against oxidative stress in in vivo and in vitro models [12,13,14,15,16].

Simulated moving bed (SMB) chromatography is a continuous countercurrent process which has been used in the separation stage for the large-scale production of compounds such as glucose and fructose [17] and chiral drugs. SMB chromatography is characterized by the separation of a few grams of thermally unstable compounds [18] and chiral drugs, which differ little in terms of their physicochemical properties [19]. Compared to semipreparative liquid chromatography, SMB requires less adsorbent and solvent, and target compounds may be derived with low loss [17,20]. Although SMB is suitable for the isolation of unstable secondary metabolites like phenolic compounds, further studies on its use are necessary. To the best of our knowledge, this study is the first to isolate and purify anthocyanins by SMB chromatography and study both their protective effects against oxidative stress and their neuroprotective ability against Aβ-induced damage in rats.

The specific aims of this study are to develop a new, highly-efficient method to isolate and purify the anthocyanins from black chokeberry, and to investigate the antioxidant activity of the SMB purified anthocyanin extract. An Aβ-induced damage animal model was used to test the effects of SMB-purified anthocyanins on spatial learning and memory ability, as well as nerve cell viability in vivo.

## 2. Materials and Methods

### 2.1. Chemicals

Cyanidin 3-*O*-glucoside standard (99%) was purchased from Sichuan Weikeqi Biological Technology CO., LTD (Chengdu, Sichuan, China) and Cyanidin 3-*O*-galactoside (Cyn-3-gal, 95%) was obtained from HaoChen Ecological Agriculture Development CO., LTD (Shanghai, China). The Aβ_1–40_ peptides were purchased from Beijing Biosynthesis Biotechnology CO., LTD (Beijing, China). 1,1-diphenyl-2-picrylhydrazyl (DPPH) and 2,2′-azino-bis(3-ethylbenzothiazoline-6-sulfonic acid) diammonium salt (ABTS), 6-hydroxy-2,5,7,8-tetramethylchroman-2-carboxylic acid (Trolox) were purchased from TCI (Shanghai) Development CO., LTD (Shanghai, China). All other organic solvents were of analytical grade.

### 2.2. Plants Material

Black chokeberry fruits were harvested at the full maturity stage in September 2017 from black chokeberry demonstration planting base in Wafangdian City (39°49′21″ N, 121°54′32″ E, Dalian, Liaoning, China). Fruits were transported at 4 °C and stored at −40 °C for a maximum of 6 months.

### 2.3. Extraction of Anthocyanins

The black chokeberry fruits were crushed and homogenized using a stainless steel blender, and then extracted twice with 65% ethanol in a 1:8 (*w*/*v*) ratio with 1% acetic acid in an ultrasonic wave bath for 10 min. Then, the supernatants were collected after centrifugation at 6485× *g* (10,000 rpm in an Anke GL-20G-Ⅱ centrifuge, Shanghai Anke company, Ltd., Shanghai, China) at 25 °C for 15 min. The supernatants were evaporated until approximately a 90% volume was achieved, and then loaded onto a column (2.6 × 60 cm) containing 100 g of the Amberlite^®^ XAD-7HP macroporous resin (Sigma Aldrich Co., St. Louis, MO, USA). The column was washed with deionized water, and then eluted with 1% acetic acid in 35% ethanol at a flow rate of 1 mL/min. Then, the crude extract solutions were evaporated, lyophilized, and stored at −40 °C.

### 2.4. Purification of Anthocyanins by SMB

**Figure 1:**
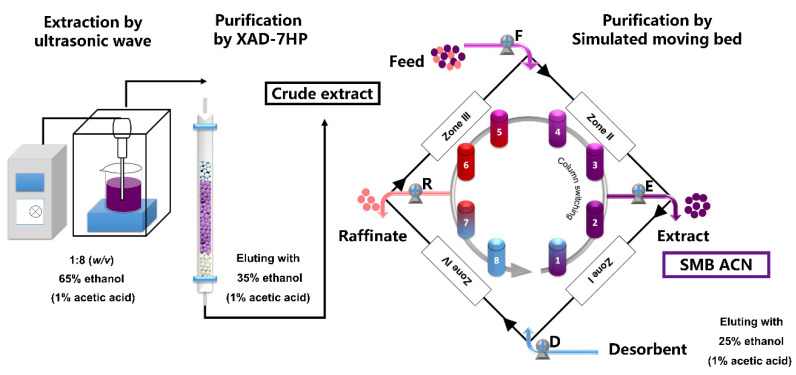
Schematic illustration of the isolation and purification of anthocyanins from black chokeberry fruits. The anthocyanins were isolated by an ultrasonic extraction method and the crude extract was prepared by XAD-7HP macroporous resins as the feed of simulated moving bed chromatography (SMB). The separation zones of SMB were zones II and III, and the solid phase was regenerated in zones I and IV with two columns per zone. The liquid phase (black) consisted of feed and desorbent for inlets, and extract and raffinate for outlets by pump E and pump R. The liquid phase was continuously fed the crude extract by pump F and eluted with ethanol by pump D. Then, SMB ACN was collected from the extract. The columns switched counter-clockwise (gray) at the switching time intervals.

Simulated moving bed chromatography (HYSMB6-500) was provided by Beijing Xiang Yue Huang Yu Technology Development Co., Ltd. (Beijing, China). The mathematical principle model is based on a rigorous mathematical first-principles model and the accurate dynamic models of multicolumn continuous chromatographic processes [18]. The anthocyanin extract solutions were homogenized by ultrasound and dissolved in 25% ethanol with 1% acetic acid at a concentration of 50 g/L through a 0.45 μm membrane to form the raw material solution used as the first SMB feed. In this SMB system (Figure 1), a four-zone working model was set up including an eluting zone (zone I), a refining (zone II) and an adsorbing zone (zone III), and a washing zone (zone IV) with two columns per zone. The SMB was equipped with eight columns (10 mm × 150 mm) with C18 (30 μm) filler configured as 2/2/2/2. The fluid was 25% ethanol with 1% acetic acid. Pump F and D pumped into the feeding and desorbent for eluting, and pump E and R pumped out the extract and raffinate. Q_E_, Q_R_, Q_D_, and Q_F_ are the flow rates of extract, raffinate, desorbent and feed, respectively. The SMB purified anthocyanin extract (SMB ACN) solutions were dried under vacuum in a rotary evaporator and lyophilized into powder, and then stored at −40 °C. HPLC-PDA was used to monitor of the extract and raffinate during the SMB process, and the purity was calculated by the area of the corresponding anthocyanin peak to the total peak area at 278 nm.

### 2.5. HPLC-PDA and UPLC-QTOF-MS Analysis

The anthocyanins extract powders were dissolved in water with 10% acetic acid and 1% phosphoric acid. Then, all samples were centrifuged at 9339× *g* (12,000 rpm) at 4 °C for 10 min, and the supernatants were used for HPLC-PDA and UPLC-QTOF-MS analysis.

The HPLC system consisted of a pump (LC-20 AT) and a photodiode array (PDA) detector (SPD-10A) (Shimadzu Corp., Tokyo, Japan). The analytical column was the Intertsil ODS-SP C18 column (4.6 × 250 mm, 5 μm, GL Sciences Inc., Tokyo, Japan). Mobile phase A was 10% acetic acid and 1% phosphoric acid in water, and mobile phase B was 100% acetonitrile. Elution was performed at a flow rate of 1.0 mL/min, and the solvent gradient was as follows: 0–8 min, 10% B; 8–12 min, B from 10% to 40%; 12–15 min, 40% B; 15–25 min, from 40% to 10% B. The injection volume was 20 μL and the column was thermostated at 25 °C. The analysis wavelengths were 278 nm for monitoring the SMB process and 520 nm for quantifying the anthocyanins [21]. The UV-VIS spectra were scanned from 220 to 800 nm. Cyanidin 3-*O*-galactoside were quantified using cyanidin 3-*O*-galactoside (*y* = 18,674*x* + 11,660, R^2^ = 0.9994) and cyanidin 3-*O*-glucoside, cyanidin 3-*O*-arabinoside and cyanidin 3-*O*-xyloside were quantified using cyanidin 3-*O*-glucoside (*y* = 25,228*x* + 10,850, R^2^ = 0.9990). The purity was determined by the area normalization method of these four anthocyanins on HPLC-PDA at 278 nm. The anthocyanin contents were determined by comparing retention times and absorption spectra, and further confirmed by UPLC-QTOF-MS.

A Waters ACQUITY UPLC system coupled with a Xevo G2QTOF mass spectrometer (Waters Corporation, Milford, MA, USA) was used for anthocyanins confirmation as previously described [22]. The separation of anthocyanins was conducted on a HSS T3 column (2.1 mm × 100 mm, 1.8 μm). The mobile phase was (A) water + 0.2% formic acid and (B) acetonitrile + 0.2% formic acid: 0 to 1 min, 5% B; 1 to 2 min, 5% to 40% B; 4 to 8 min, 95% B; 8.1 to 10 min, 5% B. The injection volume was 2 μL and the flow rate was 0.3 mL/min. The ESI parameters were set as follows: negative mode, capillary voltage 2.2 kV, sampling cone voltage 30 V, extraction cone voltage 4 V, source temperature 100 °C, desolvation gas (nitrogen) temperature 400 °C, cone gas flow rate 20 L/h, desolvation gas (nitrogen) flow rate 800 L/h, collision energies 6 eV and mass range from 50 to 1200 Da. The anthocyanin structure was determined according to its fragmentation pattern of deprotonated and product ions.

### 2.6. DPPH Free Radical Scavenging Assay

The ROS scavenging capacity was determined by the DPPH free radical scavenging assay as previously reported [23]. The assay was performed in a 96-well plate using serial dilutions of 5 μL aliquots of each Cyn-3-gal (14 to 449 mg/L), anthocyanins crude extract (2.5 to 80.0 mg/L) and SMB ACN (0.03 to 1.0 g/L). DPPH solution (200 μL, 0.06 mM) was added to each well, and the plate was incubated at room temperature in the dark for 30 min. The absorbance was determined at 520 nm using a Thermo Multiskan MK3 Automated Microplate Reader (Thermo Fisher Scientific, Waltham, MA, USA). The EC_50_ value was calculated using a calibration curve of Trolox (25 to 250 mg/L) by SPSS.

### 2.7. ABTS Free Radical Scavenging Assay

The ABTS•+ scavenging capacity assay was determined as previously described with some modifications [24]. The ABTS•+ solution was produced by reacting aqueous ABTS solution (7 mM) with potassium persulfate (2.45 mM). Diluted ABTS•+ solution with an absorbance of 0.70 ± 0.02 at 734 nm was employed in the analysis. The reactions were performed by adding 4 mL of ABTS•+ solution and 0.4 mL of each Cyn-3-gal (14 to 449 mg/L), anthocyanins crude extract (2.5 to 40.0 g/L) and SMB ACN (0.03 to 1.0 g/L). After 6 min of incubation at room temperature, absorbance values were measured on a spectrophotometer at 734 nm. The EC_50_ value of ABTS•+ scavenging capacity was calculated using a Trolox calibration curve (25 to 250 mg/L).

### 2.8. Experimental Animals

Thirty-six Sprague-Dawley (SD) rats (male, weighing 250 ± 20 g, specific pathogen-free) were purchased by Beijing HFK Bioscience Co. Ltd. (Beijing, China). The rats were kept at the animal facility with free access to standard chow and water at 22 ± 2 °C, the relative humidity of 45 ± 15% and 12 h light/dark cycle. All animals had adapted for one week before experiments. SD rats were randomly divided into 3 groups (*n* = 12) of sham, Aβ, Aβ + SMB ACN. The group of Aβ and Aβ + SMB ACN were treated by intracerebral ventricle injection of Aβ_1–40_, and then Aβ + SMB ACN group were administered by intragastric administration with anthocyanins (50 mg/kg anthocyanins 85%) for one month. The experiment was approved by the Animal Ethical and Welfare Committee (No. IRM-DWLL-2017095).

### 2.9. Preparation of Aβ-Induced Damage Rat Model

The Aβ_1–40_ oligomers were prepared according to a previously reported method [25]. Briefly, Aβ_1–40_ peptide was prepared as a stock solution at a concentration of 1 mg/mL in sterile sodium chorionic solution, followed by aggregation via incubation at 37 °C for 4 days.

Rats were anesthetized with 10% chloral hydrate (0.3 mL/100 g i.p.) and the head was symmetrically held in the stereotaxic apparatus (ZS-FD, ZS Dichuang, Beijing, China). The scalp skin was clean shaved and the skull was exposed. One hole was drilled on the skull at coordinates of −0.8 mm posterior to the bregma and 1.4 mm lateral according to the atlas of Paxinos and Watson [26,27]. The needle was injected 4 mm deep into the ventricle with a speed of 1 μL/min for 5 μL and left in place for 5 min after the injection of Aβ_1–40_. The sham rats were injected with 0.9% saline. The surgical wound was sutured and the animals were returned to their cages with free access to food and water, and allowed to recover for 1 day [28].

### 2.10. Morris Water Maze

After one month of intragastric administration with anthocyanins, the Morris water maze (MWM) test was performed as previously described [29]. The circular pool was 150 cm in diameter and 60 cm in depth with an invisible platform (12 cm in diameter and 25 cm in depth). The water in the pool was mixed with black ink. During the experiment, the temperature of the water remained within 22 to 24 °C, and all landmarks around the maze remained the same. The MWM included spatial learning and acquisition trials (hidden platform trials) and a spatial probe trial. Before the spatial acquisition trials, each rat was put into the water to adapt for 2 min and the platform was visible, located in the middle of the first quadrant for one day. Then, water was added to the pool with the platform 1 cm below the water surface. During the hidden platform trials, each rat was placed into the water from one of the start positions, facing the wall. The spatial acquisition trials were conducted over four days, with four trials per day. If the animal reached the platform, the timer was stopped. If the animal failed to find the platform within 90 s, the animal was placed on the platform for 15 s to help it learn the platform’s location. The spatial probe test was on day 5, and then the platform was removed. The rats were released from the third quadrant (180° from the original platform position) and the time spent crossing the target quadrant and the number of times the region in which the platform was previously located was crossed were recorded over a 90 s period. After testing, the rats were dried with a towel to keep them warm. The animals were recorded with a video camera, and the data were analyzed using ANY-maze behavioral tracking software (Stoelting Co., Wood Dale, IL, USA)

### 2.11. Nissl Staining

For Nissl staining of the brain, the rats were euthanized by intraperitoneal injection of 0.3% chloral hydrate, and were transcardially perfused with 100 mL of saline (0.9% *w*/*v* NaCl). Then, the brains were removed and put in the 15 mL of 4% paraformaldehyde. Serial coronal hippocampal sections with a thickness of 25 μm were cut using a cryostat (Leica Microsystems, Wetzlar, Germany). The sections were washed twice for 5 min in 0.01 M PBS and incubated in 1% toluidine blue staining solution for 5–10 min at room temperature. Then, the sections were rinsed in distilled water, soaked in 95% ethanol for 30 min, and dehydrated in 100% ethanol. After dehydration, brains were placed in xylene and cover slipped using resin medium. The neurons were quantified using image software (Image J 1.45 s), with three sections from three rats for each group.

### 2.12. Statistical Analysis

The data are presented as mean ± SD in triplicate from at least using GraphPad Prism 7.0 software (GraphPad Software, San Diego, CA, USA). *p* < 0.05 was considered statistically significant by LSD test. The results were analyzed by ANOVA and the EC_50_ value was calculated using the Probit Analysis by SPSS 20.0 software (IBM, Armonk, NY, USA).

## 3. Results

### 3.1. Isolation and Purification of Black Chokeberry Anthocyanins by SMB

**Figure 2:**
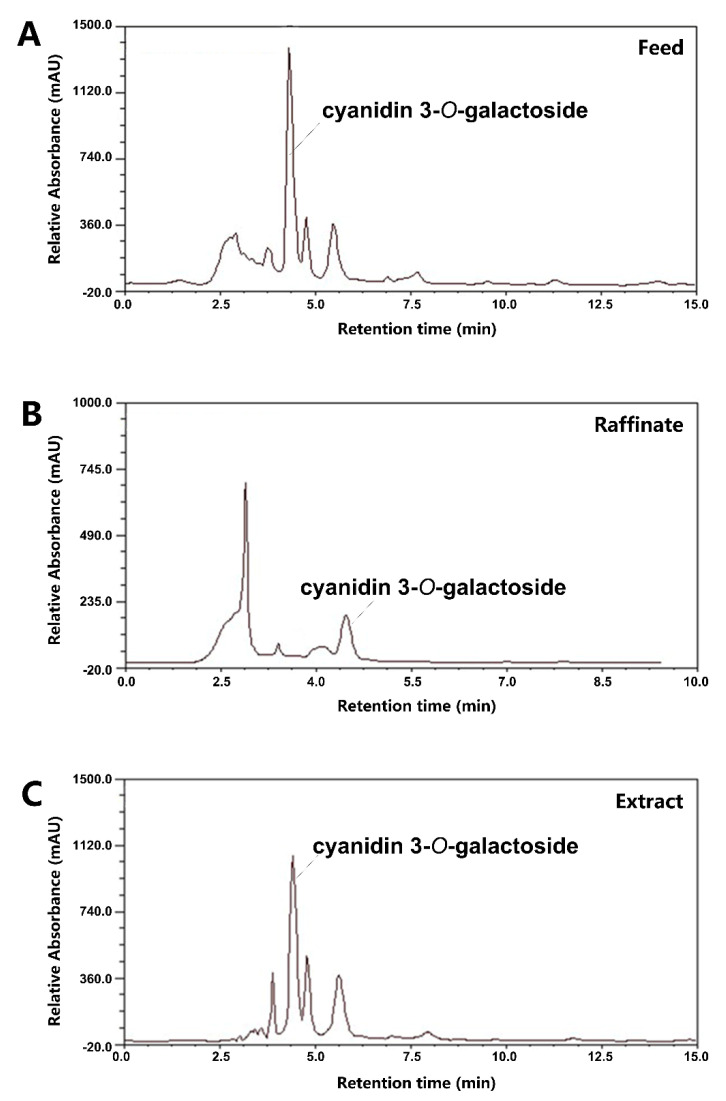
HPLC-PDA chromatogram of the feed (**A**), the raffinate (**B**), and the extract (**C**) during the SMB process monitored at 278 nm.

Anthocyanins from black chokeberry were extracted and preliminarily purified by the XAD-7HP macroporous resin column. Four-section SMB was employed to separate the anthocyanins crude extract. After approximately 20 switching times, a cyclic steady state was reached. As shown in Table 1, the optimal parameters of flow rate were Q_F_ = 0.42 mL/min, Q_E_ = 1.5 mL/min, Q_R_ = 6.5 mL/min, and Q_D_ = 4.5 mL/min. The HPLC-PDA chromatograms of feed, raffinate and extract by running H are presented in Figure 2A–C. Anthocyanins and impurities are separated in column 3 to 6 (Figure 1). The purity of the SMB ACN was estimated to be 68% to 85%. These results in run A, B and G indicated that a switching time of 141 s maximally purified the anthocyanins. At a certain switching time, the slight decrease of flow rate in the feed, extract and raffinate zone increased the purity comparing run B to C, E to G and D to H. These results confirmed that the performance of SMB was mainly associated with the switching time and flow rate of feed, extract and raffinate [30].

**Figure 3:**
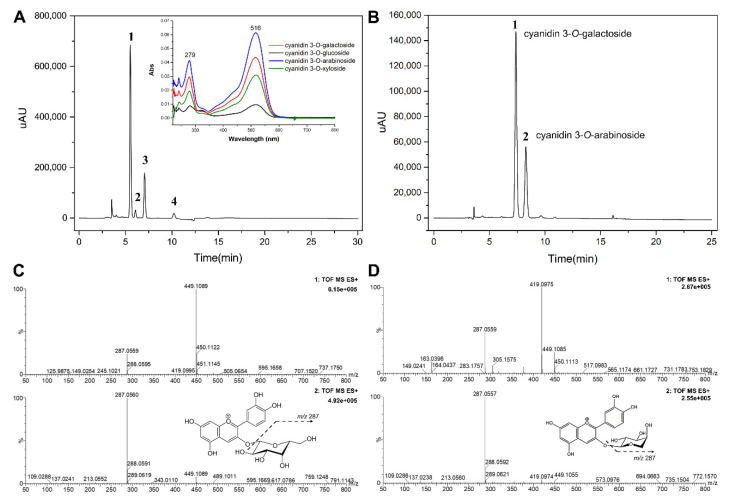
(**A**) HPLC-PDA chromatogram and UV-VIS scanning spectra of the black chokeberry anthocyanins crude extract at 520 nm and peak identities (1: cyanidin 3-*O*-galactoside; 2: cyanidin 3-*O*-glucoside; 3: cyanidin 3-*O*-arabinoside; 4: cyanidin 3-*O*-xyloside); (**B**) HPLC-PDA chromatogram of the SMB ACN at 520 nm and peak identities (1: cyanidin 3-*O*-galactoside; 2: cyanidin 3-*O*-arabinoside); The UPLC-QTOF-MS mass spectra and structure of cyanidin 3-*O*-galactoside (**C**) and cyanidin 3-*O*-arabinoside (**D**) of the SMB ACN peak 1 and peak 2.

In order to analyze the anthocyanins crude extract and SMB CAN, HPLC-PDA was used to quantify and UPLC-QTOF-MS to identify the anthocyanins. An HPLC chromatogram of the crude extract showed four peaks (Figure 3A). The λ_max_ value of UV-VIS spectra was 516 nm for cyanidin. The SMB ACN mainly showed two peaks by HPLC-PDA (Figure 3B) which were identified by UPLC-QTOF-MS (Figure 3C,D). Peak 1 of cyanidin 3-*O*-galactoside (m/z 449.1089/287.0560) appeared as a principal peak, and peak 2 was identified as a cyanidin 3-*O*-arabinoside by detection of the respective parent and product ion pairs (m/z 419.0975/287.0557). The four compounds of crude extract were cyanidin 3-*O*-galactoside (72.9% of total anthocyanins), cyanidin 3-*O*-glucoside (2.7%), cyanidin 3-*O*-arabinoside (21.0%), and cyanidin 3-*O*-xyloside (3.4%) (Table 2), which is in accordance with the Oszmianski’s results [31]. The mass of total anthocyanins in the crude extract was 2.29 ± 0.19 g/100 g of dry extract, and the mass of total anthocyanins in the SMB ACN was 61.02 ± 6.46 g/100 g of the fraction rich in dry anthocyanins.

The SMB continuously purified the anthocyanins, and efficiently enriched the two main anthocyanins and eliminated the impurity. A similar observation was made by Wang, who reported the use of a two-step simulated moving bed chromatographic process to purify the EGCG from tea polyphenol [32]. It was suggested that the SMB was a highly efficient way to separate and purify the secondary metabolites from a natural extract. Due to the instability of secondary metabolites like anthocyanins and the complexity of the plant phenols, the extraction process of monomer was extremely difficult. Therefore, these results indicated that SMB may be a feasible and effective strategy to isolate and purify the anthocyanins from black chokeberry.

### 3.2. The Free Radical Scavenging Abilities of Black Chokeberry Anthocyanin Extracts

**Figure 4:**
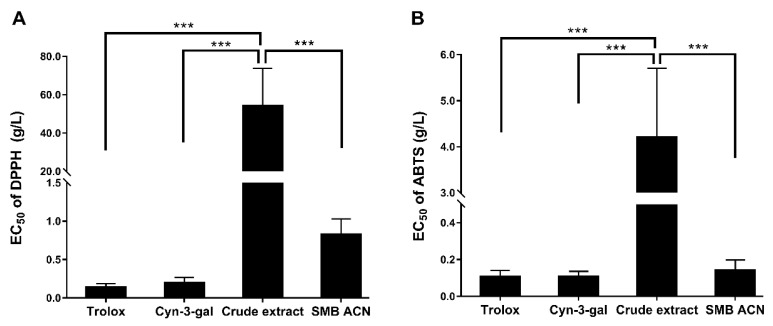
(**A**) DPPH free radical scavenging activity of Trolox, cyanidin 3-*O*-galactoside (Cyn-3-gal), anthocyanins crude extract and SMB ACN; (**B**) ABTS free radical scavenging activity of Trolox, Cyn-3-gal, anthocyanins crude extract and SMB ACN. Date represent mean ± SD (*n* = 6) were expressed by the EC_50_ inhibition of the free radical scavenging and the *** *p* < 0.001 by LSD test of one-way ANOVA.

To evaluate the SMB purification process, we tested the DPPH and ABTS free radical scavenging abilities of the black chokeberry anthocyanins crude extract and the SMB purified anthocyanin extract. As shown in Figure 4, the EC_50_ values of anthocyanins crude extract were significantly higher than Trolox, Cyn-3-gal and SMB ACN of the free radical scavenging abilities (*p* < 0.001). The DPPH• and ABTS•+ scavenging of crude extract (54.18 ± 19.59 g/L and 4.21 ± 1.50 g/L) were 65- and 30-fold more than SMB ACN (0.83 ± 0.20 g/L and 0.14 ± 0.06 g/L). The lower EC_50_ of SMB ACN indicated a higher antioxidant activity. Moreover, the ABTS radical scavenging ability of SMB ACN was similar to Cyn-3-gal and Trolox (*p* > 0.05). These results suggest that the SMB process increased the free radical scavenging ability of the anthocyanin extract.

The results showed that the ABTS free radical scavenging abilities of anthocyanins extracts were higher than those of DPPH; notably, cyanidin 3-*O*-galactoside had a high free radical scavenging ability due to its hydroxyl group. It may be that the anthocyanin extracts neutralized the DPPH free radicals mainly by hydrogen atom transfer and the ABTS•+ by a fast, non-selective electron transferring process. Therefore, the application of the SMB chromatography purified and enrichened the anthocyanins and improved the antioxidant ability than crude extract. Deneva et al. found that anthocyanins were the second biggest contributor to antioxidant activity after the proanthocyanidins among black chokeberry polyphenols [3]. Moreover, cyanidin-3-*O*-glucoside could regulate cellular antioxidant defense induced by Aβ_1–40_ in SH-SY5Y cells through the Nrf2 signaling pathway [33]. The purified anthocyanins may scavenge the free radicals and inhibit the Aβ_1–40_ neurotoxicity. Therefore, the next section of this study is concerned with SMB ACN, investigating its neuroprotective activity against amyloid-β induced in rats.

### 3.3. The Protection of Anthocyanins on Memory Impairment in Aβ-Induced Toxicity Rats

**Figure 5:**
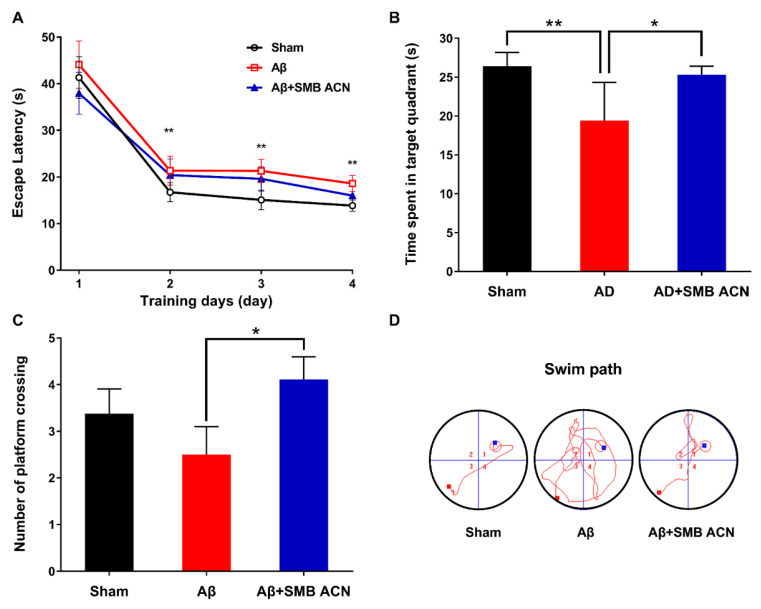
SMB ACN treatment alleviated Aβ induced spatial learning deficits. (**A**) The escape latency during the spatial acquisition trials of Sham, Aβ and Aβ + SMB ACN group; (**B**) The time spent in the target quadrant of the spatial probe test; (**C**) The number of platform crossing of the spatial probe test; (**D**) The swim path of the spatial acquisition trial. The results were expressed by mean ± SD (*n* = 12) and the * *p* < 0.05, ** *p* < 0.01 by LSD test of one-way ANOVA.

The Morris water maze (MWM) is a method to assess spatial learning and memory in rats. The escape latency time of all groups had a decreasing tendency over four days (Figure 5A). The results showed that the SMB ACN treatment group presented a less steep learning curve between days 1 and 2, which might indicate that anthocyanins had increased the short-term memory. The sham group showed a more stable spatial learning rate than the Aβ+SMB ACN group. In the probe trial, the time spent in the targeted quadrant of the sham group was shorter compared to the Aβ group impaired by Aβ_1–40_, which indicated that the Aβ group showed lower long-tern learning and memory ability (Figure 5B). The rats given the anthocyanins extract achieved more platform crossings in comparison with the Aβ-induced group, and showed a nonsignificant difference with the sham group (Figure 5C). The results indicated that anthocyanin group had a significantly higher spatial learning ability than the Aβ-induced group (*p* < 0.05). Interestingly, the sham group achieved higher scores than the Aβ group, although this was not significant, and may have been due to intervariability among the rats. As shown in Figure 5D, the Aβ + SMB ACN group allayed behavioral deficits compared to the Aβ group regarding the swim path. The behavioral test results showed that the purified anthocyanins extract alleviated the damage induced by amyloid-β to spatial learning and memory.

Previous reports have shown that anthocyanin galactosides are better maintained in the intestines compared to glucosides, while arabinosides or xylosides showed negligible losses [34]. Notably, cyanidin 3-*O*-galactoside and cyanidin 3-*O*-arabinoside crossed the blood-brain barrier into the cortex and hippocampus [16]. Moreover, the neuroprotective ability of black chokeberry has been reported; additionally, anthocyanins have been associated with antiaging [7]. Anthocyanins were shown to regulate the cell cycle and senescence by regulating the expression of the DNA damage signaling pathway and antioxidant enzymes in neuronal cells [7,11]. Our results support the hypothesis that the administration of SMB ACN ameliorates learning and memory impairment by Aβ-induced severe behavioral dysfunction.

### 3.4. The Neuroprotective Effect of SMB Anthocyanins Extract in Rat Brain

To further elucidate the neuroprotective effect of anthocyanins, we examined the hippocampus. The Nissl staining stains the nuclei of all cells, as well as clumps of material surrounding the nuclei of neurons. Neuronal loss in the brain, especially in the CA1 region of the hippocampus [35] and basal forebrain, is one of the most important pathological hallmarks of Alzheimer’s disease [36]. The pyramidal cells in the CA1 and CA3 regions of the hippocampus in the brain modulates memory and emotions [37].

**Figure 6:**
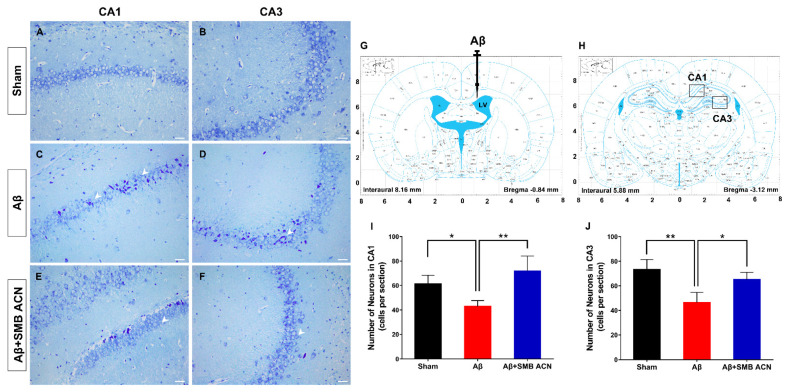
Representative images of Nissl staining in the CA1 and CA3 regions of the hippocampus of sham group (**A**,**B**), Aβ group (**C**,**D**), and Aβ + SMB ACN group (**E**,**F**). (**G**) The site of intracerebral ventricle injection in rat brain (Black) and the sites of the section in CA1 and CA3 region of the hippocampus (**H**). The number indicated the surviving neurons in CA1 (**I**) and CA3 (**J**), * *p* < 0.05 and ** *p* < 0.01. Stained sections of each group were viewed at 200× magnification. Scale bar = 100 μm.

The results showed that the survival of neuron cells in the CA1 and CA3 of the sham group was greater than in the Aβ_1–40_ treated group (*p* < 0.05) (Figure 6). The neurons in the Aβ group were disorganized and deformed with deep stain nuclei due to the neurotoxicity of Aβ. The results of the sham group indicated that the injection into the ventricle of the brain did not damage the hippocampus region. The neurons and kernel in the visual field of the Aβ + SMB ACN group were clearer and more intact than the Aβ group, and the numbers of pyramidal cells in the CA1 and CA3 were significantly increased (*p* < 0.05). The neurons in the Aβ_1–40_ treated group were damaged with extensive degenerative changes including sparse cell arrangements, swollen cell bodies, loss of integrity, shrunken cytoplasm and oval or triangular nucleus [38,39]. These results confirmed that Aβ_1–40_ can induce cellular loss and disorganization of the pyramidal cells, and indicated that the treatment of anthocyanins can reverse these changes to alleviate the loss of memory.

This research shows that purified anthocyanins by SMB exhibit excellent antioxidative activity against oxidative stress and prevent amyloid-β induced neurotoxicity in the brain. However, we still need to investigate the mechanism of antioxidative ability in vivo and the bioavailability of anthocyanins and their metabolites to determine the efficacy of such a treatment. The cell signaling pathway of anthocyanins regulating the cellular antioxidant system and alleviating the Aβ neurotoxicity still needs to be examined. We used UPLC-QTOF-MS to analyze a black chokeberry anthocyanins crude extract and found many secondary metabolites, such as polyphenols, that have antioxidant activities. In a future study, the neuroprotective ability of black chokeberry may found to be associated with the ability of these secondary metabolite compounds to protect neuronal cells against oxidative stress.

## 4. Conclusions

In this study, the anthocyanins from black chokeberry (*Aronia melanocarpa*) were isolated and purified by XAD-7HP macroporous resin and SMB chromatography, yielding a purity of 85%. The main purified anthocyanins (cyanidin 3-*O*-galactoside and cyanidin 3-*O*-arabinoside) had a strong antioxidant ability and ameliorated learning and memory impairment among Aβ-induced neurotoxicity rats. Taken together, these results demonstrate that simulated moving bed chromatography could be a feasible and effective strategy to separate highly bioactive anthocyanins from black chokeberry. The present study was an attempt to establish a method to isolate secondary metabolites from natural plants to exploit their neuroprotective abilities.

## Figures and Tables

**Table 1:** Operation parameters and separation performance of the SMB.

Run	Flow Rates (mL/min)				Switch Time ts (s)	Purity (%)
	Q_E_	Q_R_	Q_D_	Q_F_		
A	1.5	6.5	4.5	0.45	135	68.7
B	1.5	6.5	4.5	0.45	140	78.2
C	1.5	6.5	4.5	0.40	140	73.1
D	1.5	8.0	4.5	0.42	141	71.4
E	3.5	6.5	4.5	0.45	141	70.3
F	1.5	6.0	4.5	0.42	141	77.5
G	1.5	6.5	4.5	0.45	141	78.3
H	1.5	6.5	4.5	0.42	141	85.1

**Table 2:** HPLC quantification and UPLC-QTOF-MS identification of anthocyanins in Black chokeberry crude extract and SMB ACN.

Peak	RT HPLC	Compound	Molecular Formula	ESI(+)MS/MS2	Crude Extract	SMB ACN
	(min)			(m/z)	(mg/100 g FW)	(mg/g DW)
1	4.809	cyanidin 3-*O*-galactoside	C_21_H_21_O_11_	449.1089([M]+) 287.0560([M-gal]+)	500.4 ± 38.7	449.1 ± 30.8
2	5.169	cyanidin 3-*O*-glucoside	C_21_H_21_O_11_	449.1088([M]+) 287.0558([M-glu]+)	18.6 ± 1.8	ND
3	5.923	cyanidin 3-*O*-arabinoside	C_20_H_19_O_10_	419.0975([M]+) 287.0557([M-arab]+)	144.2 ± 14.0	161.2 ± 33.8
4	8.219	cyanidin 3-*O*-xyloside	C_20_H_19_O_10_	419.0975([M]+) 287.0556([M-xyl]+)	23.4 ± 1.8	ND

Data presented in fresh fruit weight (FW) and dried extract weight (DW). ND: not detected.

## Data Availability

The data are not publicly available because the research is still ongoing.
